# Ultrasensitive plasma ctDNA *KRAS* assay for detection, prognosis, and assessment of therapeutic response in patients with unresectable pancreatic ductal adenocarcinoma

**DOI:** 10.18632/oncotarget.22080

**Published:** 2017-10-26

**Authors:** Inna Chen, Victoria M. Raymond, Jennifer A. Geis, Eric A. Collisson, Benny V. Jensen, Kirstine L. Hermann, Mark G. Erlander, Margaret Tempero, Julia S. Johansen

**Affiliations:** ^1^ Department of Oncology, Herlev and Gentofte Hospital, Copenhagen University Hospital, Copenhagen, Denmark; ^2^ Trovagene, Inc., San Diego, California, USA; ^3^ Department of Medicine, University of California San Francisco, San Francisco, California, USA; ^4^ Department of Radiology, Herlev and Gentofte Hospital, Copenhagen University Hospital, Copenhagen, Denmark; ^5^ Department of Medicine, Herlev and Gentofte Hospital, Copenhagen University Hospital, Copenhagen, Denmark; ^6^ Institute of Clinical Medicine, Faculty of Health and Medical Sciences, University of Copenhagen, Copenhagen, Denmark

**Keywords:** ctDNA, KRAS, pancreatic ductal adenocarcinoma, CA19-9, prognostic biomarker

## Abstract

Precision oncology requires sensitive and specific clinical biomarkers. Carbohydrate Antigen 19-9 (CA19-9) is widely used in pancreatic ductal adenocarcinoma (PDA) but lacks sensitivity and specificity. Nearly all PDAs harbor somatic *KRAS* mutations, nominating circulating tumor DNA (ctDNA) *KRAS* as an alternative disease biomarker, however, variable clinical performance has limited its clinical utility. We applied an ultrasensitive, PCR mutation enrichment, next generation sequencing ctDNA *KRAS* assay in a large cohort of patients with unresectable PDA (*N* = 189) recruited to the BIOPAC study between 2008–2015. Baseline and longitudinal serum CA19-9 and plasma ctDNA *KRAS* were correlated with time to progression (TTP) and overall survival (OS). Baseline ctDNA *KRAS* detection rate was 93.7% (86.4% in patients with non-elevated CA19-9). ctDNA *KRAS* and CA19-9 were positively correlated yet independently associated with TTP and OS (ctDNA *KRAS p* = 0.0018 and 0.0014; CA19-9 *p* = 0.0294 and 0.0007, respectively). A generated model quantitating longitudinal ctDNA *KRAS* correctly assessed greater than 80% of patient responses. Quantitative detection of *KRAS* ctDNA is an informative prognostic biomarker, complementary to CA19-9 in patients with unresectable PDA. Longitudinal ctDNA *KRAS* may inform therapeutic decision making and provides a kinetically dynamic and quantitative metric of patient response.

## INTRODUCTION

Many of the most recent advances in oncology have been related to the field of precision medicine. For example, the introduction of targeted therapies in lung, colon, and breast cancer have contributed to a significant increase in overall survival (OS) related to these diseases [1–3]. Patients with pancreatic ductal adenocarcinoma (PDA) have not seen the same benefit of these precision oncology advances [4, 5]. The 5-year survival rate of patients with locally advanced or metastatic PDA has only seen modest increases over the last several decades, from 5% to more recent estimates of 8–9% [4–6].

Identification and utilization of reliable and informative biomarkers will play a pivotal role in precision oncology, not only for triaging patients to appropriate molecularly guided therapies, but also to inform therapeutic decision making [7]. In PDA, carbohydrate antigen 19-9 (CA19-9) has long been the biomarker of choice, has a sensitivity and specificity of 79–81% and 82–90% respectively, and is recommended for use by the National Comprehensive Cancer Network (NCCN) (v.02.2016) [8–11]. In patients with resectable disease, which accounts for less than 20% of those diagnosed [12], pre-operative serum CA19-9 levels, and serial serum CA19-9 measurements before and after surgery can be prognostic, and post-operative serum CA19-9 levels can help determine adjuvant therapy course [13]. In patients with advanced disease, the ability of serum CA19-9 to help determine treatment benefit during chemotherapy has generated conflicting results [14–20].

Currently, there are no clinically useful biomarkers established for treatment selection in patients with unresectable PDA. Daily clinical decision-making is based on imaging techniques and CA19-9. However, these tools are not always accurate in patient identification or outcome prediction. Serum CA19-9 has its limitations, primarily because 10–15% of patients do not express Lewis Body antigen, meaning that their CA19-9 levels are not informative [21]. In these patients, there is no validated biomarker for determining prognosis at time of diagnosis or for use in monitoring of therapeutic response. Additionally, CA19-9 can be elevated in non-neoplastic, inflammatory conditions, like pancreatitis, as well as due to biologic conditions like stent blockage [21, 22].

Detection of circulating tumor DNA (ctDNA) has rapidly emerged as an alternative to tissue biopsy in identifying the presence or absence of clinically actionable genomic alterations within a tumor [23–25]. Longitudinal monitoring of ctDNA has been shown to serve as a biomarker for predicting patient outcome by quantitating dynamic changes in ctDNA mutation load in response to a therapeutic intervention. Multiple studies have reported correlation between dynamic changes in the levels of a specific ctDNA mutation with radiographic response [26–28]. However, these studies have examined individual patients (i.e., anecdotal data) and not the development and testing of a clinical tool within a patient population that could be used to monitor and predict response. PDA presents a unique opportunity for molecular diagnosis and monitoring given that over 90–95% of patients with PDA harbor somatic *KRAS* G12/G13 mutations as the primary driver of mutagenesis [29–32]. Given this fact, measurement of ctDNA for detection and quantitative monitoring of *KRAS* mutations may offer a complementary diagnostic and prognostic biomarker to CA19-9. Researchers have attempted to harness advancing ctDNA technologies to understand the clinical utility of ctDNA *KRAS* in patients with PDA as a diagnostic and prognostic biomarker, biomarker for drug response, and molecular monitoring tool. Previous studies have reported low ctDNA *KRAS* detection rates ranging from 27% to 71%, hampering the advancement of ctDNA *KRAS* as a reliable clinical tool [23, 27, 33–38].

Here we report on the application of an ultrasensitive ctDNA *KRAS* assay used in patients with unresectable PDA, and its use as a prognostic biomarker independent and complementary to CA19-9. We also introduce novel work utilizing dynamic changes in ctDNA *KRAS* mutation load from serial measurements as an assessment of therapeutic response.

## RESULTS

In this prospective, retrospective analysis of CA19-9 and ctDNA *KRAS* in patients with newly diagnosed American Joint Committee on Cancer (AJCC) stage III or stage IV PDA, 189 patients met inclusion criteria and had baseline samples that passed preliminary quality control analysis (Table 1). The majority of patients (78.8%) were diagnosed with Stage IV disease. In this cohort, all patients were treated with chemotherapy with the primary first line chemotherapy being gemcitabine (66.7%) which is consistent with primary therapy for this patient population during the recruitment period. Median number of ctDNA *KRAS* timepoints per patient was 3 (interquartile range (IQR) 2–4) and median number of CA19-9 timepoints per patient was 8 (IQR 4–16).

**Table 1:** Demographics for 189 patients

Characteristic	Median (Q1–Q3)
**Age**, years	67 (60–72)
	**Number (%)**
**Gender**	
Male	95 (50.3)
Female	94 (49.7)
**ECOG Performance Status**	
0	70 (37.4)
1	99 (52.4)
2	20 (10.6)
**Stage**	
III	40 (21.2)
IV	149 (78.8)
**Chemotherapy type**	
Gemcitabine	126 (66.7)
FOLFIRINOX	63 (33.3)
**ctDNA *KRAS* mutation detected at baseline^1^**	
G12A	4 (2.3)
G12C	18 (10.2)
G12D	72 (40.7)
G12R	22 (12.4)
G12S	4 (2.3)
G12V	55 (31.1)
G13D	2 (1.1)

^1^Available for 177 patients.

Q - quartiles.

### Baseline statistics and primary variates

**Figure 1:**
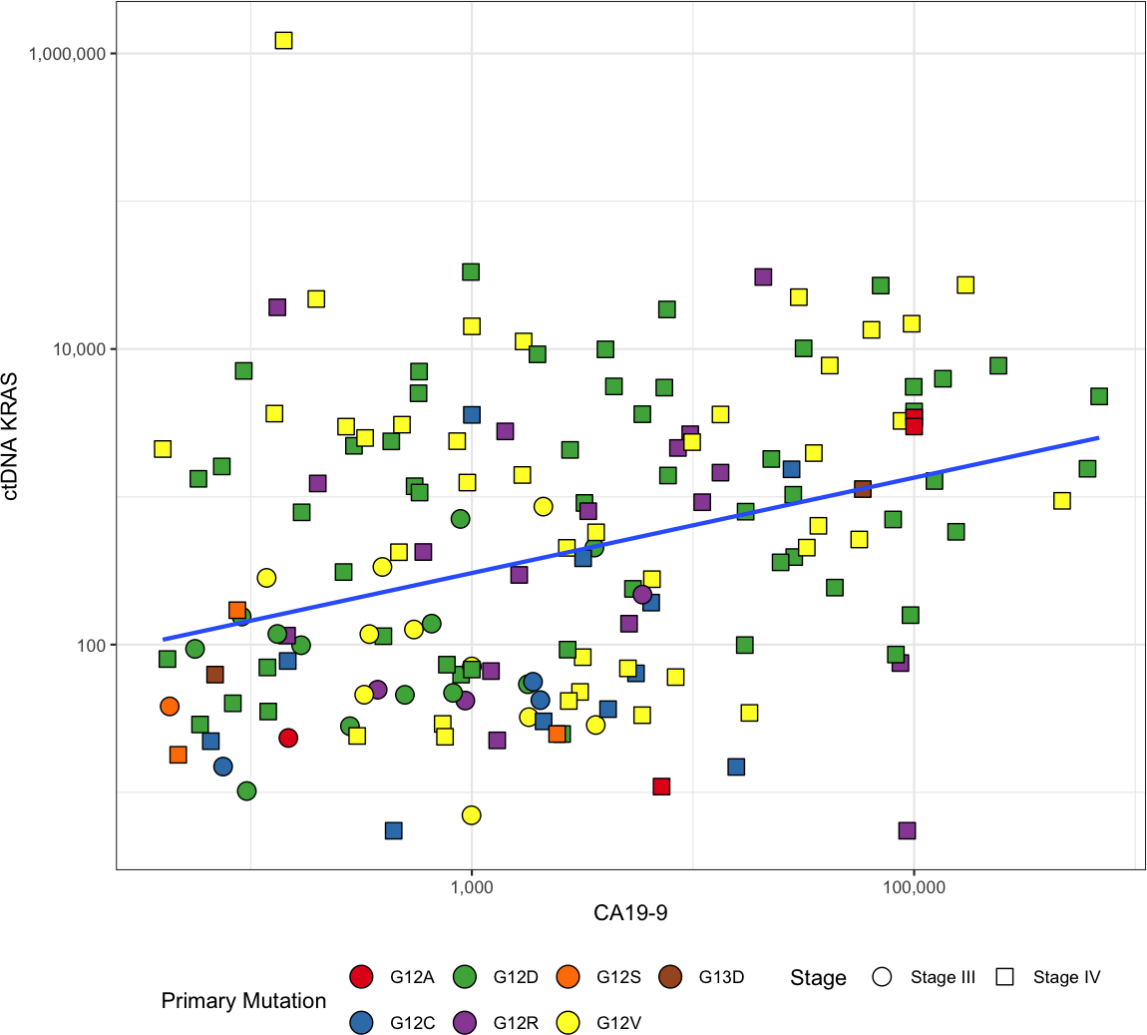
Significant positive relationship between baseline CA19-9 and ctDNA *KRAS* for positive samples (*N* = 158) Pearson's correlation coefficient *r* = 0.295; *p* = 0.0002. Spearman's Rank Correlation *r* = 0.312; *p* < 0.0001.

Plasma ctDNA *KRAS* mutation detection rate in this 189 patient cohort was 93.7% (*N* = 177; 95% confidence interval (CI) 89.2%–96.7%) (Table 2). The most frequently detected *KRAS* mutations were G12D (40.7%) and G12V (31.1%) (Table 1), which is consistent with the most common mutations identified through somatic testing of pancreatic adenocarcinoma tumors [39]. The median number of ctDNA *KRAS* copies detected at baseline was 335.4 copies per 10^5^ Genome Equivalents (GEq) (IQR of 54–2,214 copies per 10^5^ GEq). For CA19-9, 167 patients had baseline levels above 37 units per milliliter (U/mL) and were considered elevated (88.4%; 95% CI 82.9%–92.6%) (Table 2). The median U/mL was 1,890 (IQR 412 – 12, 160 U/mL). ctDNA *KRAS* was detectable at baseline for 19 of 22 patients that had non-elevated CA19-9 levels (86.4%; 95% CI 65.1%–97.1%) (Table 2). The addition of baseline ctDNA *KRAS* to this cohort significantly increased patient identification (*p* = 0.0020). For baseline positive samples, there was a significant, positive relationship between CA19-9 and ctDNA *KRAS* (Figure 1) (Pearson's Correlation Coefficient *r* = 0.295; *p* = 0.0002; Spearman's rank correlation *r* = 0.312; *p* < 0.0001).

**Table 2:** Detection concordance between ctDNA KRAS and serum CA19-9

CA19-9				
		Detected	Not Detected	Total
**ctDNA *KRAS***	Detected	158	19	177
	Not Detected	9	3	12
	Total	167	22	189

ctDNA – circulating tumor DNA.

**Figure 2:**
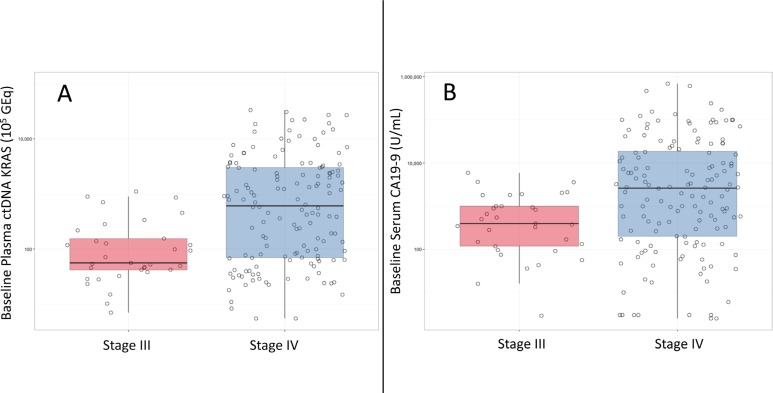
Boxplots of ctDNA *KRAS* (**A**) and CA19-9 (**B**) for baseline detected samples.

**Figure 3:**
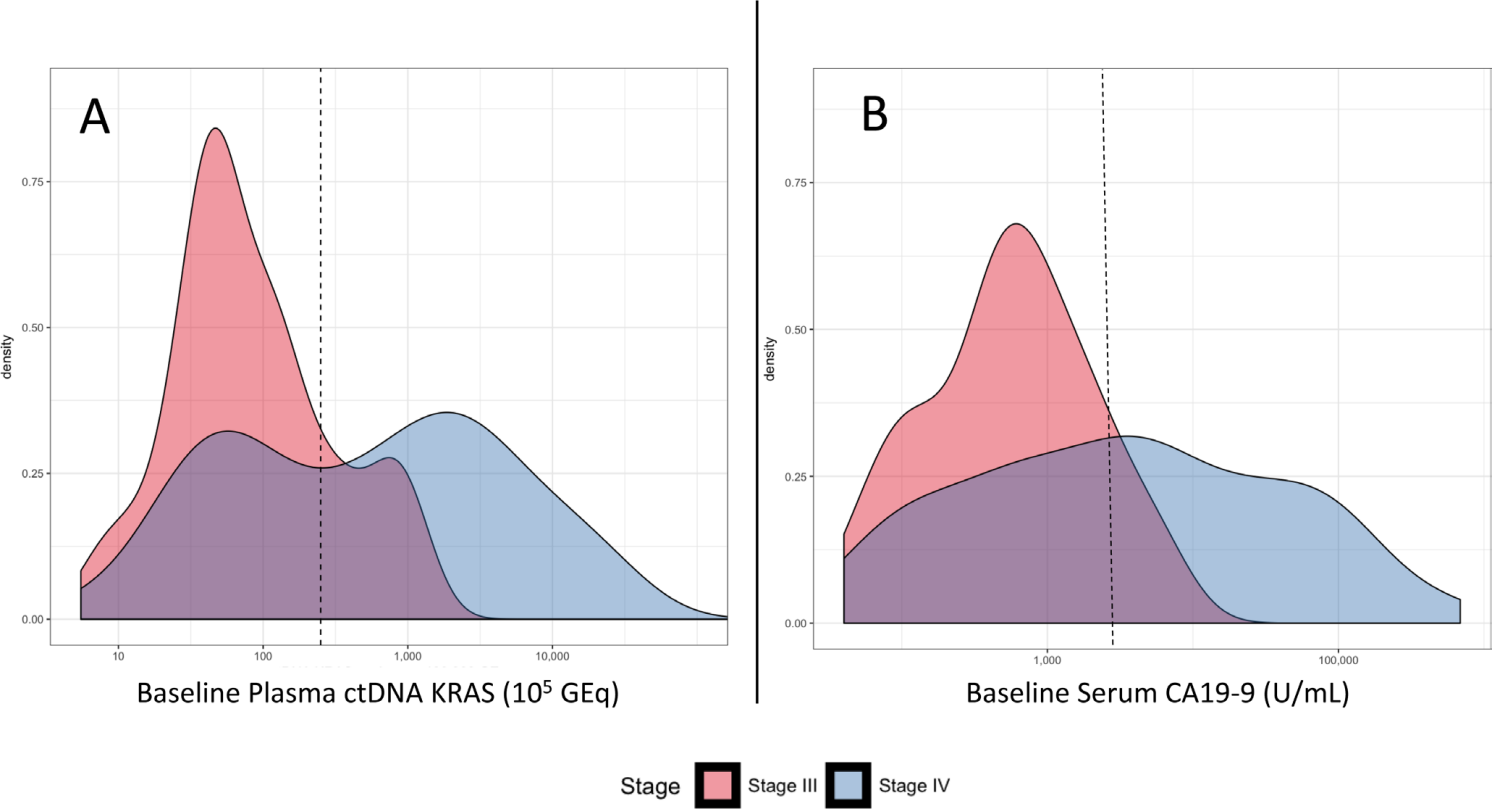
(**A**) Density plot of ctDNA *KRAS* copies per 10^5^ GEq in 177 patients with detectable baseline results stratified by stage. Dotted vertical line denotes the bimodal cutpoint of approximately 250 copies per 10^5^ GEq. (**B**) Density plot of CA19-9 U/mL for 167 patients with detected baseline samples stratified by stage. Dotted vertical line denotes the median cutpoint of approximately 1,890 units per milliliter.

The median number of baseline plasma ctDNA *KRAS* copies was higher in patients with stage IV versus stage III PDA (608.2 versus 56.0 copies per 10^5^ GEq; *p* < 0.0001) (Figure 2A). This was also observed in baseline serum CA19-9 (3,230 versus 540 U/mL; *p* < 0.0001) (Figure 2B). When the quantitative baseline values of ctDNA *KRAS* and CA19-9 were plotted versus disease stage, there was a notable bi-modal distribution for ctDNA *KRAS* that was not observed for CA19-9 (Figure 3). The resulting natural cut-point was approximately 250 ctDNA *KRAS* copies per 10^5^ GEq (Figure 3A). This natural cut-point was utilized in subsequent calculations to categorize the number of ctDNA *KRAS* copies into “High” and “Low” bins. For serum CA19-9, the median (1,890 U/mL) was utilized in subsequent calculations for a similar categorization (Figure 3B).

**Figure 4:**
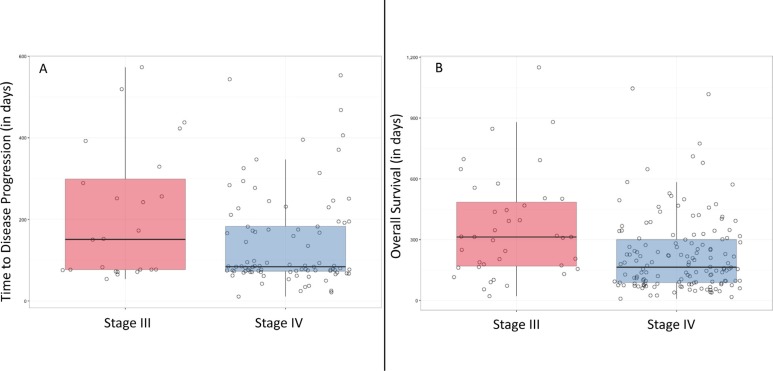
Boxplots of time to disease progression in days (**A**) and overall survival in days (**B**) for baseline samples stratified by stage.

### Association with time to disease progression

One-hundred and thirteen patients achieved disease progression (PD) during first line of palliative chemotherapy (59.8%; 95% CI 52.4%–66.8%). Primary reasons for not reaching PD were death and loss to follow-up (missing CT evaluation). Median time to progression (TTP) during first line of chemotherapy was 85 days and did not differ significantly based on disease stage (stage IV: 84 days versus stage III: 151 days; *p* = 0.1094) (Figure 4A). However, there was a difference in TTP for stage IV versus III disease that trended towards significance (Hazard Ratio (HR) = 1.26, 95% CI 1.00–1.59, *p* = 0.0504). Irrespective of disease stage, baseline ctDNA *KRAS* above the natural bimodal cutpoint was associated with PD (HR 1.29; 95% CI 1.06–1.57, *p* = 0.0105), as was CA19-9 above its respective median (HR 1.25; 95% CI 1.02–1.53, *p* = 0.0354).

In a univariate analysis, baseline ctDNA *KRAS* was associated with TTP (HR = 1.42; 95% CI 1.12–1.76, *p* = 0.0013). In a multivariate regression analysis, utilizing baseline CA19-9, and baseline ctDNA *KRAS* as continuous variables, along with the co-variates of interest, stage, gender (sex), and baseline European Cooperative Oncology Group (ECOG) performance score, ctDNA *KRAS* remained highly significant for TTP (HR = 1.45; 95% CI 1.15–1.83, *p* = 0.0017), while baseline CA19-9 was less significant (HR = 1.27; 95% CI 1.03–1.58, *p* = 0.0284) along with sex (HR = 0.79; 95% CI 0.63–0.99, *p* = 0.0400) (Table 3).

**Table 3:** Multivariate regression analysis: prognostic performance of continuous variable ctDNA *KRAS* and CA19-9

	Variate	HR (95% CI)	Coefficient*p*
Overall Survival^*^			
	CA19-9	1.30 (1.11–1.53)	0.0014
	ctDNA *KRAS*	1.45 (1.17–1.80)	0.0007
	Sex	0.79 (0.67–0.93)	0.0060
	Age	1.03 (1.01–1.05)	0.0068
	Performance Score 1 and 2	0.80 (0.67–0.96)	0.0148
Time to Progression^*^			
	CA19-9	1.27 (1.03–1.58)	0.0284
	ctDNA *KRAS*	1.45 (1.15–1.83)	0.0017
	Sex	0.79 (0.63–0.99)	0.0400

^*^ Overall model fit was assessed by Likelihood and Wald scores. Corresponding *p*-values of the model were < 0.0001 indicating a good fit.

**Figure 5:**
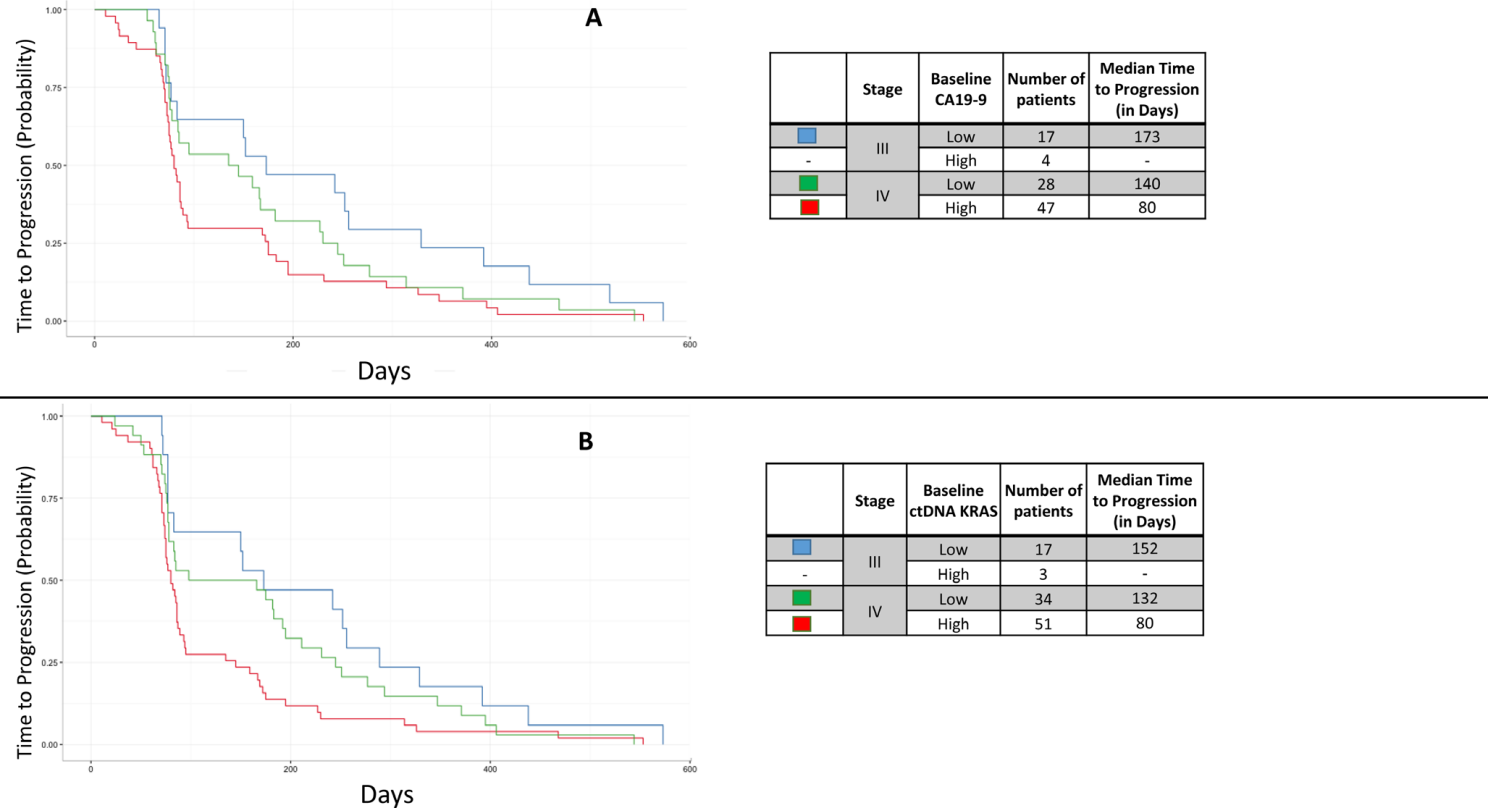
Kaplan Meier curves demonstrating the patient time to progression (TTP) by stage and categorical baseline CA19-9 (**A**) and categorical baseline ctDNA *KRAS* status (**B**). High/low CA19-9 status was based on the median in this cohort (1,890 U/mL) while high/low ctDNA *KRAS* status was based on the bimodal cutpoint of 250 copies per 10^5^ Genome Equivalents. Patients with high baseline values of ctDNA *KRAS* had significantly shorter TTP than those with low baseline values (*p* = 0.0358) which was not appreciated in high/low CA19-9 status (*p* = 0.1237). The number of patients with Stage III disease and high baseline CA19-9 was too few for calculation, as was the number of patients with Stage III disease and high baseline ctDNA *KRAS*.

When analyzing the categorical cutpoints and TTP, there was not a significant difference in the median TTP in patients with stage IV disease based on their baseline CA19-9 level (*p* = 0.2550; Figure 5A). In contrast, those with a diagnosis of stage IV disease had a borderline significant association based on their ctDNA *KRAS* level (*p* = 0.0554; Figure 5B). The number of patients with stage III disease and high baseline CA19-9 (*N* = 4) was too few for calculations, as was the number of patients with stage III disease and high baseline ctDNA *KRAS* (*N* = 3).

### Association with overall survival

OS was calculated for 183 patients for a median OS of 188 days (IQR of 96–326 days). Patients with stage IV disease had a significantly shorter OS than those with stage III disease (stage IV: 164 days versus stage III: 313 days; *p* = 0.0005) (Figure 4B). In addition to this, a baseline ctDNA *KRAS* above the natural bimodal cut-point (Hazard ratio (HR) = 1.36; 95% CI 1.16–1.59, *p* = 0.0001), a baseline CA19-9 value above the median (1,890 U/mL) (HR = 1.34; 95% CI 1.15–1.57, *p* = 0.0003), and disease stage (stage III versus stage IV) (HR = 1.34; 95% CI 1.12–1.61, *p* = 0.0014) were each significantly associated with OS.

Univariate analysis demonstrated that baseline plasma ctDNA *KRAS* as a continuous variable (HR = 1.42; 95% CI 1.19–1.69, *p* < 0.0001) and baseline serum CA19-9 as a continuous variable (HR = 1.36; 95% CI 1.17–1.59, *p* < 0.0001) were each directly associated with OS significantly. In a multivariate regression analysis, categorical ctDNA *KRAS* and CA19-9 along with the other co-variates (stage, gender (sex), baseline ECOG performance status) were utilized to determine their respective associations with OS. Both ctDNA *KRAS* and CA19-9 when analyzed with the co-variates of interest were associated with OS (ctDNA *KRAS*: HR = 1.45; 95% CI 1.17–1.80, *p* = 0.0007; CA19-9: HR = 1.31; 95% CI 1.11–1.53, *p* = 0.0014). Other significant variates included gender and baseline ECOG performance status (Table 3).

**Figure 6:**
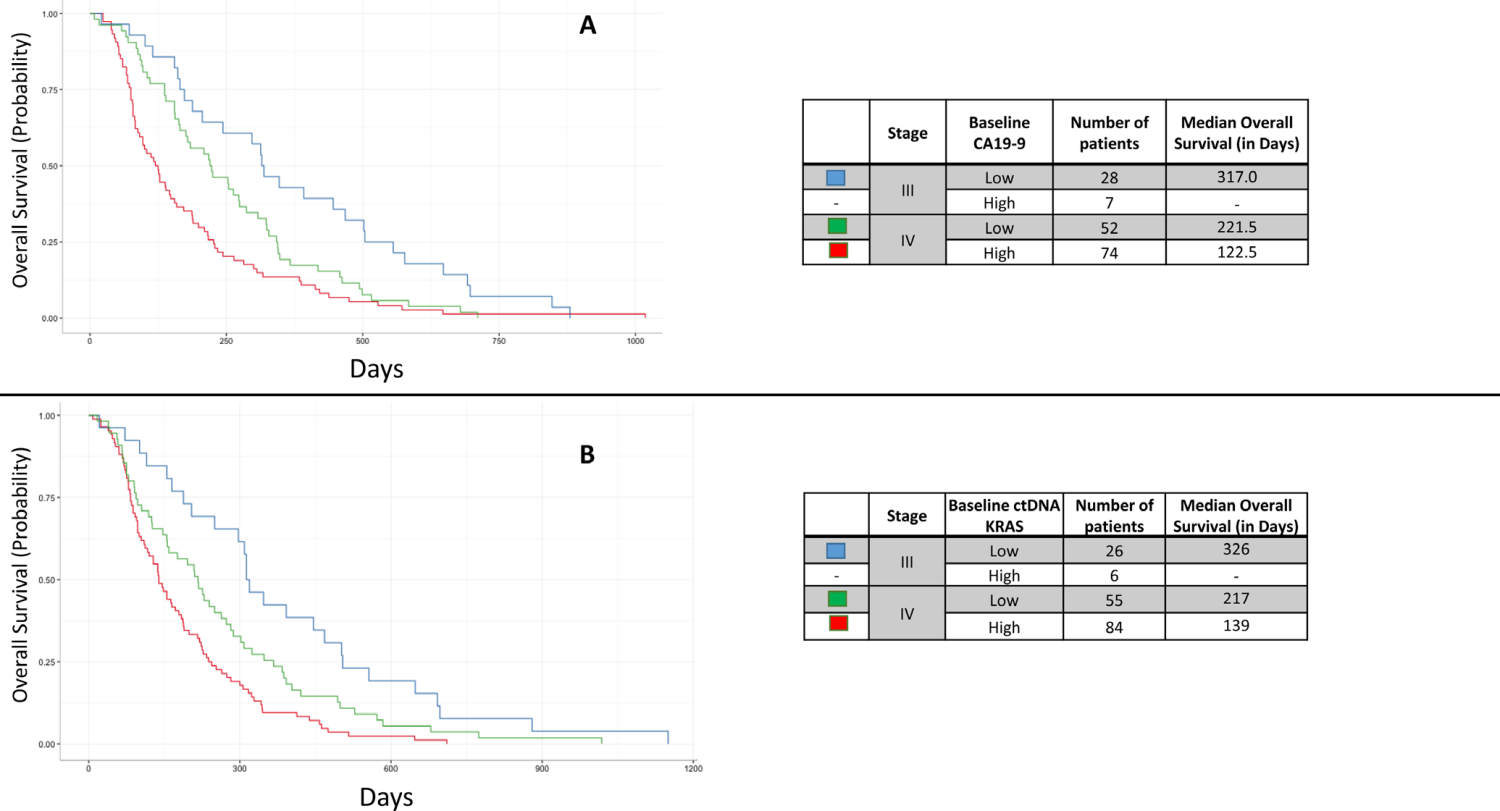
Kaplan Meier curves demonstrating the overall survival (OS) by stage and categorical baseline CA19-9 (**A**) and categorical baseline ctDNA *KRAS* status (**B**). High/low CA19-9 status was based on the median in this cohort (1,890 U/mL) while high/low ctDNA *KRAS* status was based on the bimodal cutpoint of 250 copies per 10^5^ Genome Equivalents. Patients with high baseline values had significantly shorter OS than those with low baseline values. The number of patients with Stage III disease and high baseline CA19-9 was too few for calculation, as was the number of patients with Stage III disease and high baseline ctDNA *KRAS*.

Patients with stage III disease and low baseline CA19-9 (*N* = 28) had a longer OS than those with stage IV disease, regardless of which categorical CA19-9 status (*p* < 0.0001 for CA19-9 high, *p* = 0.0333 for CA19-9 low; Figure 6A). Patients with stage IV disease and a high baseline CA19-9 level (*N* = 74) had a significantly shorter OS than those with stage IV disease and a low baseline CA19-9 (*N* = 52; *p* = 0.0009) (Figure 6A). The number of patients with stage III disease and high baseline CA19-9 was too few for calculations (*N* = 7). Similarly, for baseline ctDNA *KRAS*, patients with stage III disease and low baseline ctDNA *KRAS* levels (*N* = 26) had a longer OS than patients with stage IV disease, regardless of baseline ctDNA *KRAS* level (*p* = 0.0170 and < 0.0001; Figure 6B). Patients with stage IV disease and a high baseline ctDNA *KRAS* (*N* = 85) had a significantly shorter OS than those with stage IV disease and low baseline ctDNA *KRAS* levels (*N* = 55; *p* = 0.0425) (Figure 6B). The number of patients with stage III disease and high baseline ctDNA *KRAS* was too few for calculation (*N* = 6), as was the number of patients with Stage III disease and high baseline CA19-9 (*N* = 7).

### Association with performance status

**Figure 7:**
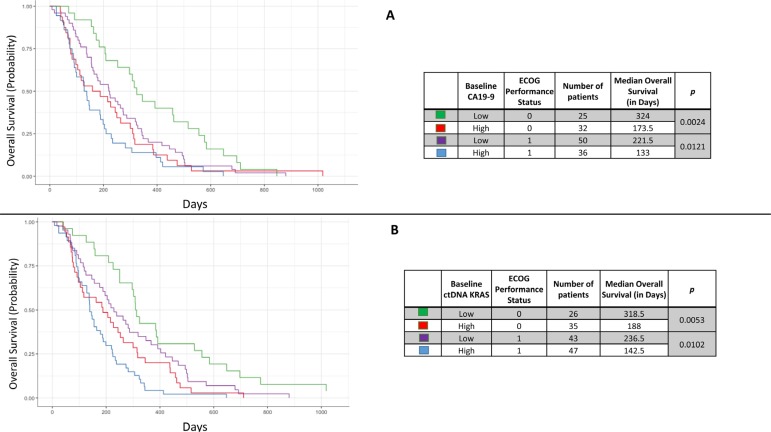
Kaplan Meier curves demonstrating the overall survival based on categorical CA19-9 (high/low) and pretreatment ECOG performance score (**A**) and similarly, the overall survival based categorical ctDNA *KRAS* (high/low) and pretreatment ECOG performance score (**B**).

Association of OS with the patient's pre-treatment ECOG performance status was examined with baseline high and low ctDNA *KRAS* and CA19-9 levels. In patients with a performance status of 0, there was a statistically significant difference in OS between those patients with high versus low baseline CA19-9 levels (*p* = 0.0024). This also held true in patients with a performance status of 1 (*p* = 0.0121) (Figure 7A). The differences in OS were also significant for those with a performance status of 0 and high versus low baseline ctDNA *KRAS* (*p* = 0.0053) as well as a performance status of 1 (*p* = 0.0102) (Figure 7B).

### Plasma ctDNA *KRAS* as a response biomarker

**Figure 8:**
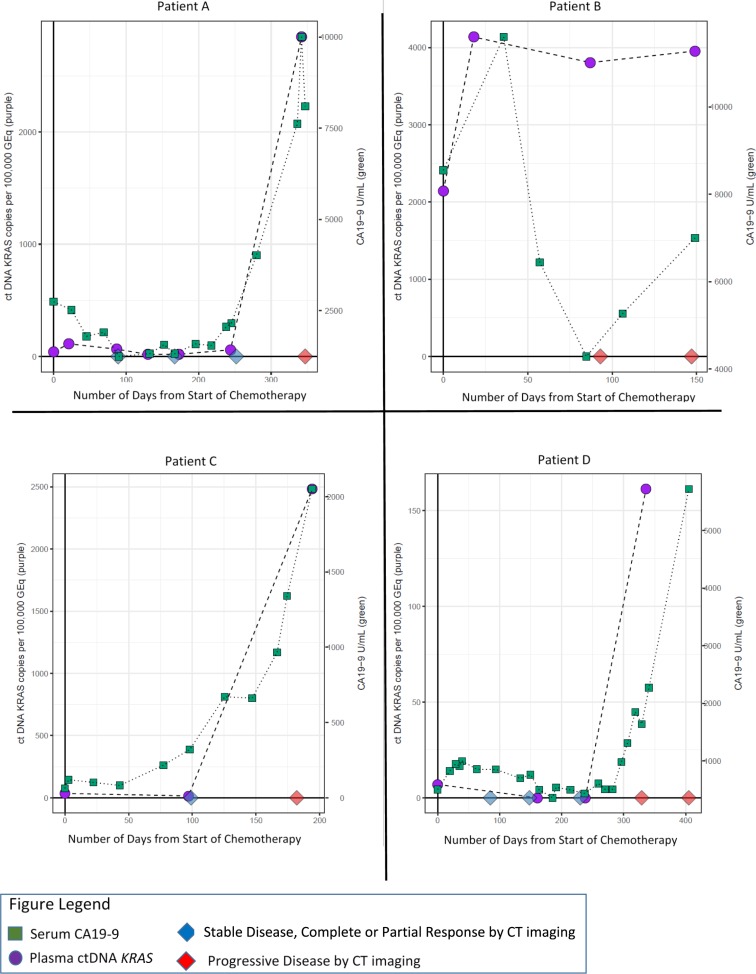
Representative longitudinal plots of patients who had a different investigator assessed response as compared to the confirmatory RECIST 1.1 criteria Green squares represent longitudinal CA19-9 collection timepoints, while purple circles represent longitudinal ctDNA *KRAS* collection timepoints. The CT scan RECIST 1.1 assessment is represented by diamonds on the X-axis. CT scans are truncated to include only those that were within 2 weeks of a ctDNA *KRAS* collection.

In a subset of patients, details about investigator assessed progression were available in addition to CT scan response by RECIST1.1, and ctDNA *KRAS* and CA19-9 data (*N* = 88 of 113 patients who reached PD on first line therapy). Most patients had an investigator assessed response that was confirmed by RECIST 1.1 criteria (*N* = 62 of 88; 70%). To determine whether dynamic changes in longitudinal ctDNA *KRAS* levels were consistent with RECIST 1.1 assessment, patients in which there was a plasma ctDNA *KRAS* within 2 weeks of a RECIST 1.1 assessment were examined. 51.6% of these patients (*N* = 32) had a ctDNA *KRAS* collection within 2 weeks of a RECIST 1.1 assessment, and dynamic longitudinal changes in mutation load trended consistent with RECIST 1.1 assessment in 23 patients (71.9%). In the remaining 26 patients where the investigator assessment of response differed from RECIST 1.1, 20 patients had a ctDNA *KRAS* longitudinal timepoint within 2 weeks of a RECIST 1.1 assessment. We observed that longitudinal monitoring of changes in ctDNA *KRAS* mutational burden trended consistent with RECIST 1.1 response in nearly half of these patients (*N* = 9 of 20; Figure 8).

**Figure 9:**
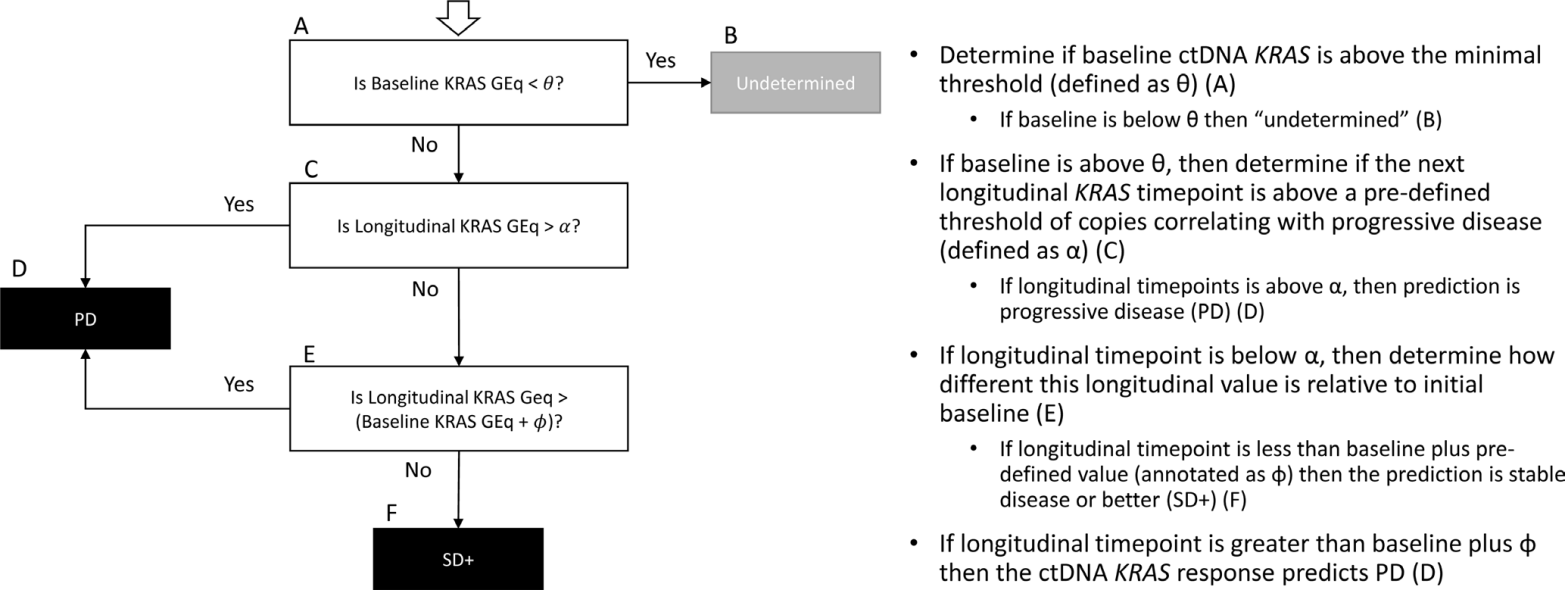
Predictive response model logic

**Figure 10:**
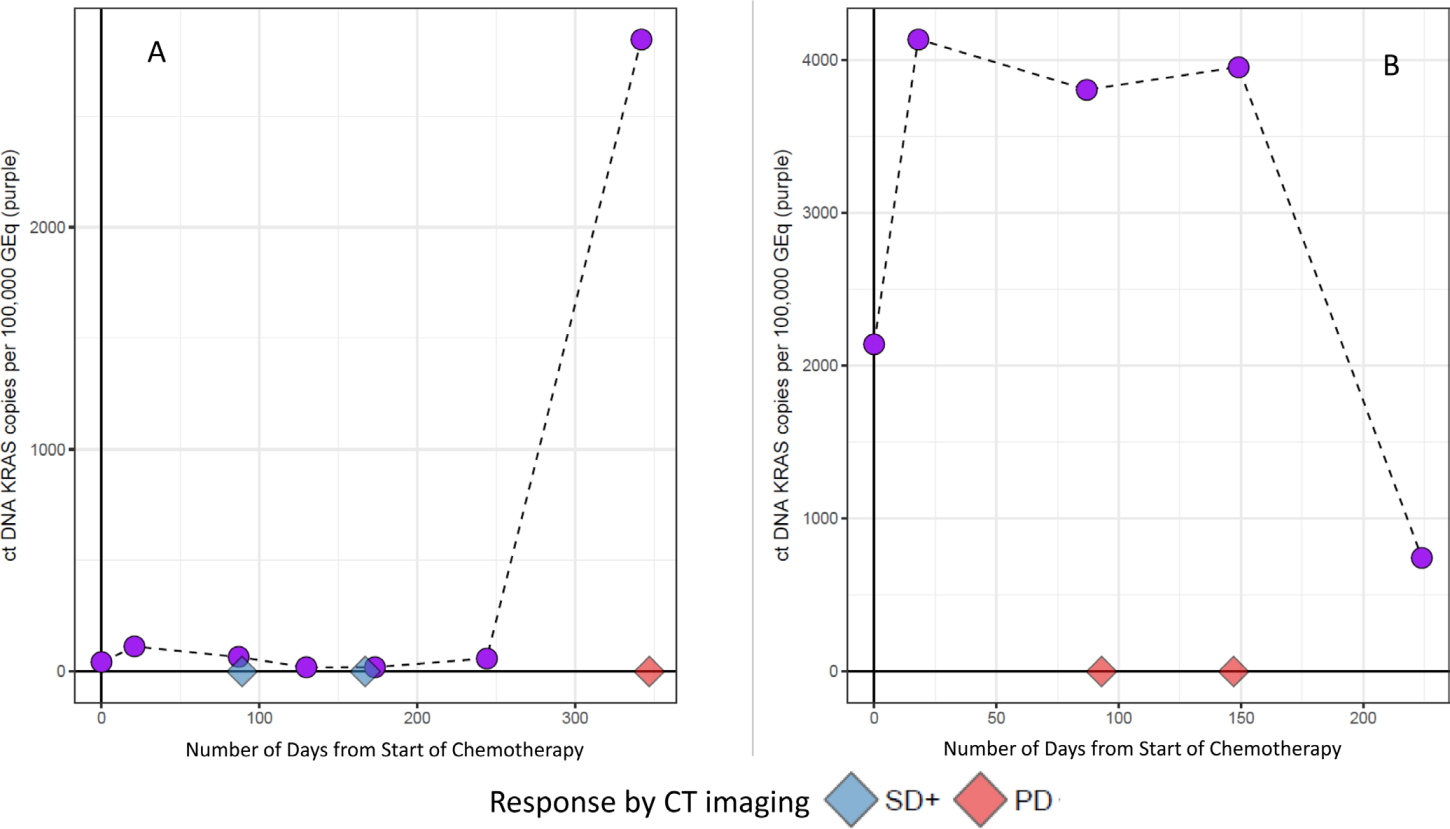
Representative longitudinal plots of patients in whom the ctDNA *KRAS* predictive model correctly assessed the RECIST 1.1 response The CT scan RECIST 1.1 assessment is represented by diamonds on the X-axis. CT scans are truncated to include only those that were within 7 days of a ctDNA *KRAS* collection.

**Figure 11:**
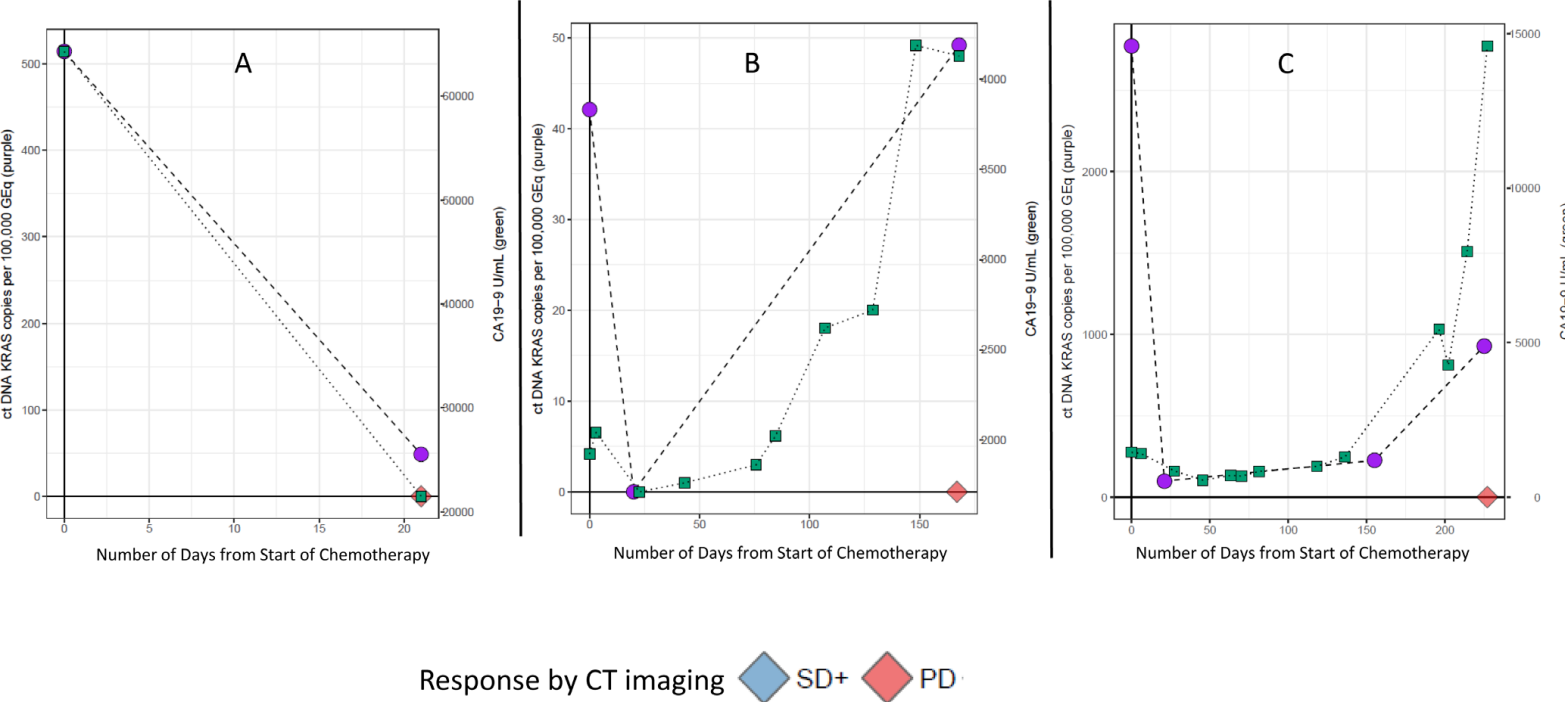
Discrepant predictive modeling plots Patient A had decreasing CA19-9 and ctDNA KRAS levels as compared to baseline, but a RECIST 1.1 assessment of progressive disease (PD) (**A**). Patient B had increasing CA19-9 and ctDNA KRAS as compared to baseline, but the difference between the baseline and longitudinal timepoints did not meet threshold for a PD call (**B**). In Patient C, there was a predicted response of stable disease response, and the longitudinal ctDNA KRAS was just below our threshold, thus this is a borderline case (**C**).

These observations were further explored and formalized with predictive modeling (Figure 9). Of the dataset, there were 59 timepoints (matched baseline and longitudinal timepoint) from 46 unique patients that met criteria for predictive modeling (one matched timepoint (*N* = 37), two matched timepoints (*N* = 6), three matched timepoints (*N* = 2) and four matched timepoints (*N* = 1). Four of the 59 matched timepoints were “undetermined” as they had a baseline ctDNA *KRAS* GEq that was less than θ. For the remaining 55 samples, utilizing the quantitative baseline ctDNA *KRAS* level and the longitudinal quantitative ctDNA *KRAS* level that was within 7 days of a verified RECIST 1.1 timepoint, the model correctly predicted the RECIST 1.1 results (PD versus SD+) in 81.8% of matched timepoints (*N* = 45 of 55; 95% CI 69.7%–90.9%) (Figure 10). Four of the 10 samples with discrepant calls had an associated longitudinal timepoint that was “not detected” for ctDNA *KRAS*, therefore the model predicted a response of “SD+” while the RECIST 1.1 response was PD. In the remaining six discrepant matched samples the model predicted a response of SD+, but actual response was PD. In one matched sample ctDNA *KRAS* value was borderline as the longitudinal timepoint was just below the threshold (Figure 11) and in two matched samples the ctDNA *KRAS* longitudinal trend that was mirrored by CA19-9 (Figure 11).

## DISCUSSION

We report on the utilization of a novel mutant allele enrichment ctDNA assay in patients with advanced PDA yielding a circulating tumor DNA (ctDNA) *KRAS* baseline detection rate of 93.7% (95% CI 89.2%–96.7%), outperforming the detection rate of prior reported assays [23, 27, 33–38]. The percentage of individuals without an informative biomarker was reduced from 11.6% when utilizing CA19-9 alone (22 of 189 patients), to 1.6% when utilizing both serum CA19-9 and plasma ctDNA *KRAS* (3 of 189 patients). Utilization of CA19-9 and ctDNA *KRAS* in this cohort increased the yield of positively identified patients by 10.1% and resulted in a positive biomarker result in 186 of 189 patients. ctDNA *KRAS* and CA19-9 were significantly positively correlated, demonstrating good performance of ctDNA *KRAS* alongside the currently accepted standard of care biomarker for PDA.

In univariate analysis, both baseline CA19-9 and ctDNA *KRAS* were associated with overall survival (OS) which was expected given the known positive correlation between the two variates. In multivariate analysis both CA19-9 and ctDNA *KRAS* were significantly associated with OS, indicating that these two biomarkers capture unique, but complementary prognostic information. The multivariate analysis suggests that assessment of ctDNA *KRAS* and CA19-9 levels could be used jointly to achieve greater prognostic certainty within the clinical setting.

Baseline ctDNA *KRAS* was associated with time to progression (TTP). In a multivariate analysis with CA19-9 and the additional co-variates (stage, gender, baseline ECOG performance status), ctDNA *KRAS* remains highly significant while CA19-9 was less significant.

We observed a natural bimodal distribution of baseline quantitative plasma ctDNA *KRAS* copy number when analyzed versus PDA disease stage. Upon exploration of this bimodal distribution and natural cutpoint, it was confirmed that there are significant differences in outcome (OS, TTP) between these two patient populations, irrespective of stage of disease. However, when ctDNA *KRAS* was examined within predefined stage III or stage IV disease, individuals with high versus low baseline ctDNA *KRAS* copies also had a significantly different OS and TTP. These two observations indicate that assessment of plasma ctDNA *KRAS* levels for an individual patient may give greater clarity of prognosis than disease stage alone and underscores the potential clinical impact of ctDNA *KRAS*, a molecular biomarker, to further sub-classify prognosis beyond the current methodologies. Patients with PDA and poorer performance status and low baseline ctDNA *KRAS* levels had a longer OS than those patients with a better performance status and high baseline ctDNA *KRAS* levels.

Assessment of response to chemotherapy is challenging in patients with advanced PDA, as evidenced by the cohort of patients where in 30% of cases the investigator assessed response differed from response as confirmed by RECIST 1.1 criteria. In PDA, specifically, given the lethality of the cancer, there is the strong desire for the ability to rapidly ascertain response and inform the decision of whether to continue an effective therapeutic regimen or discontinue an ineffective one [40].

The utilization of serum CA19-9 has been explored as a potential biomarker of response, but with conflicting results [14, 41, 42]. A study of more than 200 patients with metastatic and locally advanced PDA concluded that changes in CA19-9 levels should not be used to make decisions about discontinuing or continuing therapies given the lack of significant positive predictive value. The authors suggest that a more rigorous analysis, limiting the time collection timepoints to within the first two cycles of chemotherapy could be clinically meaningful in treatment decision making [20].

Since the first report in 1948 by Mandel and Metais, describing the presence of cell-free nucleic acid in human blood, ctDNA and its clinical utility have been of clinical interest [43–45]. Assessment of ctDNA, termed as “liquid biopsy”, can provide complementary dynamic information, which can aid in clinical decision making [24]. Somatic *KRAS* mutations have been reported to be present in greater than 90–95% of PDA cases assessed by tumor tissue analysis suggesting a potential biomarker in detecting and monitoring disease progression [29–32]. Several studies have been published demonstrating the presence of ctDNA in plasma in patients with PDA with varying sensitivity [23, 27, 33–37, 46–51]. Yamada *et al.* reported that high plasma ctDNA *KRAS* was positively correlated with stage and that persistent post-treatment detectable ctDNA *KRAS* mutations was associated with poorer prognosis [46]. Recently, Kinugasa *et al.* utilized a droplet digital PCR ctDNA technology and identified ctDNA *KRAS* mutations in 62.6% of patients with PDA (stages II, III and IV). Patients with detectable ctDNA *KRAS* mutations had significantly shorter survival than patients without detectable mutations [52]. Pietrasz *et al*. reported that 41.3% of patients with advanced PDA had detectable ctDNA *KRAS* mutation by NGS and droplet-based dPCR in microfluidics with a high concordance between the two technics [38].

The high prevalence of somatic *KRAS* mutations in PDA provides an opportunity to have an informative, specific biomarker of response that may aid in assessment of response, identifying patients who are not likely to benefit from additional cytotoxic chemotherapy. In a novel predictive modeling algorithm, examining the quantitative change between ctDNA *KRAS* mutation load at baseline (pre-treatment) and at subsequent CT scan correctly predicted the RECIST 1.1 assessment in approximately 82% of matched timepoints. This analysis was restricted to timepoints collected while on first line therapy, and those timepoints collected within 7 days of a RECIST 1.1 assessment. The ability of longitudinal monitoring to correctly assess the current disease state would provide clinicians with a rapid, non-invasive assessment of response that could be repeated frequently. Future analysis will expand this modeling in a larger cohort of patients with PDA and with more structured collection timepoints and intervals. We observed that longitudinal monitoring of changes in ctDNA *KRAS* trended consistent with RECIST 1.1 response in nearly half of the patients. It would also be interesting in future studies to evaluate the correlation between ctDNA *KRAS* and tumor load or shrinkage, as well as whether the depth of decrease of *KRAS* ctDNA from baseline could be predictive for survival.

The current study has several strengths. First, the study population was treated and followed within a single hospital system, allowing accurate clinical metric follow-up. Second, the Danish universal health care system allowed for detailed tracking of death dates for the patients. Third, this is one of the largest, if not the largest study of the application of ctDNA in patients with unresectable PDA.

However, the study is not without its limitations. First, matched tumor tissue *KRAS* analysis was not available for this cohort. It is widely accepted that more than 90–95%, of PDAs harbor somatic *KRAS* G12/G13 mutations. We relied on this background knowledge, along with the mutation distribution within the cohort that was consistent with what was expected, to estimate that the ultrasensitive ctDNA *KRAS* assay detected almost all samples correctly, however “tissue truth” was not available. Second, this analysis was retrospectively applied to prospectively collected samples. While the BIOPAC study design outlined the sample collection timepoints, these were not uniformly applied. This impacted the number of plasma ctDNA timepoints available within a RECIST 1.1-assessed CT scans. Future prospectively designed studies will have more rigorous enforcement of study sample collection timepoints and intervals.

In conclusion, plasma ctDNA *KRAS* was detected in 93.7% of patients with stage III/IV PDA. The addition of plasma ctDNA *KRAS* to serum CA19-9 at baseline significantly increased patient identification. High baseline ctDNA *KRAS* mutation levels were significantly associated with OS and TTP in both univariate and multivariate models and were more significantly associated with prognosis than CA19-9. Longitudinal monitoring with ctDNA *KRAS* as a response biomarker successfully predicted response in over 80% of patients with PDA. The utilization of this ultrasensitive ctDNA *KRAS* assay not only provides an informative prognostic biomarker, but may also aid in clinical decision making at diagnosis and during treatment.

## MATERIALS AND METHODS

### Patients

Patients diagnosed and histologically verified with AJCC Stage III or Stage IV unresectable PDA between July 2008 and August 2015 at Herlev and Gentofte Hospital were eligible for the Danish BIOPAC (BIOmarkers in Pancreatic Cancer) Study and were prospectively enrolled [53]. The study is ongoing and approved by the Regional Ethics Committee (VEK ref. KA-20060113) and the Danish Data Protection Agency (j.nr. 2006-41-6848 and HGH-2015-027, I-Suite nr: 03960). Informed consent for research use was obtained from all patients at the enrolling institution (Herlev and Gentofte Hospital, Copenhagen University Hospital, Denmark) before prospective plasma banking.

Patients were qualified for inclusion if they had a diagnosis of PDA, confirmed with CT scan and histology/cytology and were of age 18 years or older at the time of diagnosis. Patients were treatment naïve at the time of enrollment and were treated with first line gemcitabine or fluorouracil (5-FU), leucovorin, irinotecan, and oxaliplatin (FOLFIRINOX). During the study period, 241 patients were recruited to the study, 52 of whom were excluded for the following reasons: no baseline (pre-treatment) sample received (*N* = 10), no baseline (pre-treatment) CT scan (*N* = 11), data was from second line of chemotherapy (*N* = 2), patient had resectable disease (*N* = 2), patient was diagnosed with non-pancreas cancer (*N* = 2), patient was treated with radiation (*N* = 1), and ctDNA assay failed (*N* = 24) leaving 189 patients for analysis (Table 1). During treatment CT evaluation was missed in 76 patients due to cancer related death or rapid clinical deterioration. In some cases the data collection intervals were irregular due to different schedules of chemotherapy (Gemcitabine 3 weeks cycle vs. FOLFIRINOX 2 weeks cycle), accelerated sample collection because of accelerated CT scan or missing samples. Last follow-up was August 2016.

### Sample collection and processing

Baseline and longitudinal plasma samples were collected on all enrolled patients when possible. Study design outlined plasma collection timepoints as baseline (pre-treatment, prior to initiation of first line of chemotherapy), before administration of the second cycle of first line of chemotherapy, and thereafter with every CT scan. Actual collection time-points varied. Biobanking strategy in all enrolled patients was designed with the same standard operating procedures for the acquisition, handling and storage of blood samples. Plasma samples were collected in sodium citrate tubes and centrifuged within ½–2 hours (2330 g for 10 min at 4°C), and stored at −80°C with temperature alarm. 1.2–2 mL of plasma ctDNA was isolated using the QIAamp Circulating Nucleic Acid kit (Qiagen, Valencia, CA) according to the manufacturer's instructions.

### Clinical data collection

Medical records and outcome data of all included patients were collected and reviewed retrospectively. Changes in tumor burden on the consecutive scans were assessed according to the RECIST 1.1 by an independent radiologist, blinded to all clinical data and ctDNA *KRAS* information. In 30% of cases the investigator assessed response differed from response as confirmed by RECIST 1.1 criteria. One-hundred and thirteen patients achieved disease progression as assessed by RECIST criteria. Thus, appearance of undoubtedly new lesions was classified as progressive disease (PD). We followed RECIST guidelines in case of a new lesion was equivocal. If repeated scans confirmed definitely a new lesion, then progression date was stated using the initial scan.

### Plasma ctDNA *KRAS* analysis

Quantitative analysis of the seven most common mutations in *KRAS* exon 2, codons 12 and 13 (G12A, G12C, G12D, G12R, G12S, G12V, G13D) was performed using a mutation-enrichment polymerase chain reaction (PCR) coupled with next generation sequencing (NGS) utilizing an ultra-short footprint PCR assay (gene specific footprint 31 bp, with overall amplicon length of 75 bp) to amplify highly degraded ctDNA *KRAS* fragments [54].

A proprietary pipeline processed the FASTQ files from the Illumina's MiSeq Sequencing Platform with algorithms that were validated with a sensitivity of 96.0% (95% CI of 86.3%–99.5%) and specificity of 97.9% (95% CI of 88.7%–99.9%) with samples obtained from healthy volunteers, patients with wild-type *KRAS* G12/G13 cancer and patients with mutant *KRAS* G12/G13 cancer. Given that detectable *KRAS* G12/G13 mutation levels were identified, the pipeline then accurately quantifies the mutational load using a corresponding reference set of standard samples that are processed on the same run. Final mutation copy numbers were scaled to 10^5^ Genome Equivalents (GEq, refers to amount of DNA in a single cell) [54]. The concordance was 94% between *KRAS* G12/G13 mutations in plasma cell-free DNA and FFPE tumor tissue [54].

### Serum CA19-9 analysis

Pretreatment and longitudinal serum CA 19-9 concentration was analyzed using the Immulite 2000 GI-MA (Siemens, Catalogue Number L2KG12) assay, a solid-phase, two-site sequential chemiluminescent immunometric assay. The upper normal limit for serum CA 19.9 was 37 U/mL, as this is the reference range for CA19-9.

### Statistical analysis

The results of this project are reported in accordance with the REMARK (Reporting recommendations for tumor marker prognostic study) guidelines [55]. Results were compiled for the 189 patients with available baseline, pre-treatment ctDNA specimens successfully processed through the proprietary pipeline along with the demographic information collected at baseline, CT imaging scans assessed with RECIST 1.1 criteria and serum CA19-9 results taken throughout treatment. At each timepoint, specimens with ctDNA *KRAS* G12/G13 quantitative copy numbers greater than the established lower limit of detection were labeled as “detected”. At each timepoint, specimens with CA19-9 levels with greater than or equal to 37 U/mL were considered “elevated” according to the CA19-9 reference range. Analyzed longitudinal plasma and serum samples were collected after baseline, prior to the start of the second line of chemotherapy (when occurred) and at each RECIST 1.1 assessed CT imaging within two weeks of collection.

The response end points examined were overall survival (OS) and Time to Progression (TTP). OS is defined as the number of days from the start of the first line of chemotherapy to death. TTP is defined here as the number of days from the start of the first line of chemotherapy to PD given that progression is achieved prior to the start of any subsequent lines of chemotherapy. PD is defined using the RECIST 1.1 criteria as have a greater than 20% increase in sum of the diameters of target lesions with an absolute increase of ≥ 5 mm [56].

DNA analyses were performed retrospectively and independently from the clinical data review. Predictive modeling was implemented in 46 patients, in which ctDNA *KRAS* level was within 7 days of a verified RECIST 1.1 timepoint. Statistical analysis examined both primary variates of interest, ctDNA *KRAS* copies per 10^5^ GEqand CA19-9 U/mL, against each other and with the response end points. Relationships for both response end points and primary variates along with co-variates of age, gender (sex), chemotherapy type, stage, and pre-treatment ECOG performance status were evaluated. Non-significant variates were removed from consideration using backwards selection.

Cox proportional hazards regression models were used to assess the significance of the variates of interest. Confidence intervals were assessed and reported at 95% unless otherwise stated, and significance testing was two-sided with *p* < 0.05 considered statistically significant. Due to the high skewness of the data, a log10 transformation was applied to both plasma ctDNA *KRAS* copy number and serum CA19-9 prior to analysis in order to preserve the normality assumption. The model was tested for fit utilizing Wald and Likelihood scores.

### Predictive model for response

Additional analyses were performed for those samples with two or more plasma collection timepoints and RECIST 1.1 CT scan data available. Analysis was restricted to patients with a detectable baseline ctDNA *KRAS* result and at least one subsequent longitudinal ctDNA timepoint within 7 days of a CT scan with RECIST 1.1 assessment. RECIST 1.1 response was simplified and reported as PD or SD+. SD+ includes a RECIST 1.1 assessment of stable disease, partial response, or complete response [56].

First, we assessed the longitudinal trends in quantitative ctDNA *KRAS* mutation load and correlated with RECIST 1.1 data and investigator assessed response. The trends observed between longitudinal measurements of ctDNA *KRAS* and RECIST 1.1 status were strong enough to be explicitly quantified and demonstrated the ability to identify RECIST 1.1 response by measuring quantitative changes in ctDNA *KRAS* mutation load as compared to baseline. Given a minimal threshold of ctDNA *KRAS* copies at baseline (annotated as θ), trends were analyzed over large search grids to determine the minimum ctDNA *KRAS* copies correlating with ctDNA *KRAS* response of PD (annotated as α). If a longitudinal measurement was less than the α minimum PD threshold and less than the baseline measurement plus a corresponding shift threshold (annotated as ϕ), the ctDNA *KRAS* response was assessed as SD+. Otherwise, the ctDNA *KRAS* response was called to be PD (Figure 5).

All statistical analyses were carried out using R version 3.2.3 statistical software.

EAC has received consulting fees from Qiagen and Guardant and research support from Ignyta, Daiichi-Sankyo.

## Abbreviations

BIOPAC: Biomarkers in Pancreatic Cancer
CA19-9: Carbohydrate Antigen 19-9
ctDNA: circulating tumor DNA
CI: confidence interval
GEq: Genome Equivalents
HR: Hazard ratio
IQR: interquartile range
NGS: next generation sequencing
OS: overall survival
*p*: *p* value
PCR: polymerase chain reaction
PD: disease progression
PDA: Pancreatic Ductal Adenocarcinoma
RECIST: Response Evaluation Criteria In Solid Tumors
SD: Stable disease
TTP: time to progression
U/mL: units per milliliter.
